# Honeybee Exposure to Veterinary Drugs: How Is the Gut Microbiota Affected?

**DOI:** 10.1128/spectrum.00176-21

**Published:** 2021-08-11

**Authors:** Loredana Baffoni, Daniele Alberoni, Francesca Gaggìa, Chiara Braglia, Catherine Stanton, Paul R. Ross, Diana Di Gioia

**Affiliations:** a Department of Agricultural and Food Sciences (DISTAL), University of Bologna, Bologna, Italy; b Teagasc Food Research Centre, Moorepark, Fermoy, County Cork, Ireland; c APC Microbiome Institute, University College Cork, County Cork, Ireland; Broad Institute

**Keywords:** *Bombilactobacillus*, *Lactobacillus*, next-generation sequencing, NGS, antibiotic resistance, bifidobacteria, honeybees, microbiota, sulphonamides, tetracyclines, tylosin

## Abstract

Several studies have outlined that a balanced gut microbiota offers metabolic and protective functions supporting honeybee health and performance. The present work contributes to increasing knowledge on the impact on the honeybee gut microbiota of the three most common veterinary drugs (oxytetracycline, sulfonamides, and tylosin). The study was designed with a semi-field approach in micro-hives containing about 500 honeybees. Micro-hives were located in an incubator during the day and moved outdoors in the late afternoon, considering the restrictions on the use of antibiotics in the open field but allowing a certain freedom to honeybees; 6 replicates were considered for each treatment. The absolute abundance of the major gut microbial taxa in newly eclosed individuals was studied with qPCR and next-generation sequencing. Antimicrobial resistance genes for the target antibiotics were also monitored using a qPCR approach. The results showed that the total amount of gut bacteria was not altered by antibiotic treatment, but qualitative variations were observed. Tylosin treatment determined a significant decrease of α- and β-diversity indices and a strong depletion of the rectum population (lactobacilli and bifidobacteria) while favoring the ileum microorganisms (*Gilliamella*, *Snodgrassella*, and *Frischella* spp.). Major changes were also observed in honeybees treated with sulfonamides, with a decrease in *Bartonella* and *Frischella* core taxa and an increase of *Bombilactobacillus* spp. and *Snodgrassella* spp. The present study also shows an important effect of tetracycline that is focused on specific taxa with minor impact on alfa and beta diversity. Monitoring of antibiotic resistance genes confirmed that honeybees represent a great reservoir of tetracycline resistance genes. Tetracycline and sulfonamides resistance genes tended to increase in the gut microbiota population upon antibiotic administration.

**IMPORTANCE** This study investigates the impact of the three most widely used antibiotics in the beekeeping sector (oxytetracycline, tylosin, and sulfonamides) on the honeybee gut microbiota and on the spread of antibiotic resistance genes. The research represents an advance to the present literature, considering that the tylosin and sulfonamides effects on the gut microbiota have never been studied. Another original aspect lies in the experimental approach used, as the study looks at the impact of veterinary drugs and feed supplements 24 days after the beginning of the administration, in order to explore perturbations in newly eclosed honeybees, instead of the same treated honeybee generation. Moreover, the study was not performed with cage tests but in micro-hives, thus achieving conditions closer to real hives. The study reaches the conclusion that the most common veterinary drugs determine changes in some core microbiota members and that incidence of resistance genes for tetracycline and sulfonamides increases following antibiotic treatment.

## INTRODUCTION

Bees have a globally recognized importance for the maintenance of plant biodiversity and for pollination of crops (1, 2). In addition, honeybees are appreciated for the production of commercially important hive products, such as honey, propolis, royal jelly, and wax (3, 4).

Several biotic and abiotic factors have contributed to the honeybee decline observed in the last 20 years in western countries (United States and European Union) (5, 6). The intensive agricultural systems, with the use of pesticides and weed killers, have determined scarcities of foraging resources for honeybees (7). However, the greatest threat to honeybee survival are pathogens and parasites that have spread widely at a global scale, favored by intensive honeybees rearing practices (8) such as the close proximity of honeybees hives (9) and exchange of honeybees among different colonies (10). In this way, honeybees can no longer survive without constant anthropogenic inputs (4, 11) in many regions worldwide.

In order to fight microbial pathogens, several antibiotics have been used, such as oxytetracycline-HCl (Terramicin) against Paenibacillus larvae (12), tylosin (Tylovet) against Melissococcus plutonius (13, 14), and sulfonamides to control both pathogenic bacteria and, partially, nosemosis caused by Nosema apis and Nosema ceranae (14).

The use of antibiotics has promoted the spread of antibiotic resistance genes among pathogenic and commensal bacteria, which has led many nations to apply restrictions on their use on livestock (15, 16). In the beekeeping sector, most of the authorizations to trade certain antibiotics have been withdrawn by the European Commission or by pharmaceutical companies themselves (17, 18). Conversely, antibiotic administration to honeybees is permitted in many other countries, though with restriction and controls (19, 20), and the European honey market is still threatened by antibiotic residues (21).

Several recent works have outlined the effects of antibiotic use on the honeybee gut microbiota (22–24). The honeybee gut microbiota is relatively simple, composed of a few core bacterial genera and other non-core genera with a low or occasional presence (25, 26). Commensal gut bacteria, in addition to their role in honeybee nutrition and physiology, act in synergy with the host immune system and play a role in modulating the insect response to pathogens (27, 28). The honeybee gut microbiota is directly influenced by various factors, such as diet, season, and exposure to chemical compounds such as weed killers or antibiotics (22, 29, 30), and its unbalance, defined as intestinal dysbiosis (31), may negatively influence honeybee well-being.

In this work, we investigated the effect on the honeybee gut microbial community of the most widely used veterinary drugs oxytetracycline, sulfonamides, and tylosin. Few studies, often based on cage tests or on a hybrid approach consisting of a cage test followed by a short time of reintroduction into the honeybee colony, have considered the impact of oxytetracycline on the honeybee gut microbiota, whereas, to the best of our knowledge, sulfonamides and tylosin have never been investigated before. This study has been performed using a semi-field approach, e.g., in experimental conditions as close as possible to real hives considering the restrictions on the use of antibiotics, thus partially avoiding artificial conditions typical of the cage tests. Perturbation of the gut microbiota in newly eclosed individuals were explored with the use of quantitative PCR (qPCR) and next-generation sequencing (NGS). In addition, antimicrobial resistance genes for the target antibiotics were monitored.

## RESULTS

### General observations on the colony’s status pre- and posttreatment.

The trial involved bees treated with tetracycline (Pan-Terramicina, PT), sulfonamides (Sulfac, SUL) and tylosin (Tylan, TL), plus an untreated control (CTR); each experimental condition was tested with 6 replicates. Bees were sampled at T0 (experiment beginning) and T1 (24 days later). Moreover, the experiment relied on micro-hives managed with a semi-field approach due to national restriction on antibiotics.

Throughout the trial, no particular changes or deficiencies in the health status of the treated honeybees were observed. Only one micro-hive collapsed (PT_6) just after the experiment’s end, presumably due to varroosis, whereas CTR_5, PT_1 and SUL_1 were found to be queenless at the end the experiment. Visual evaluation at the time of gut sampling highlighted a reddish coloration of the intestinal epithelium in the tylosin treatment group. Drought conditions in the second half of the experiment did not allow nectar harvest and consequently no weight increase was observed, despite the sugar syrup supplementation.

### qPCR quantification of target microbial groups in the gut and resistance genes.

**FIG 1:**
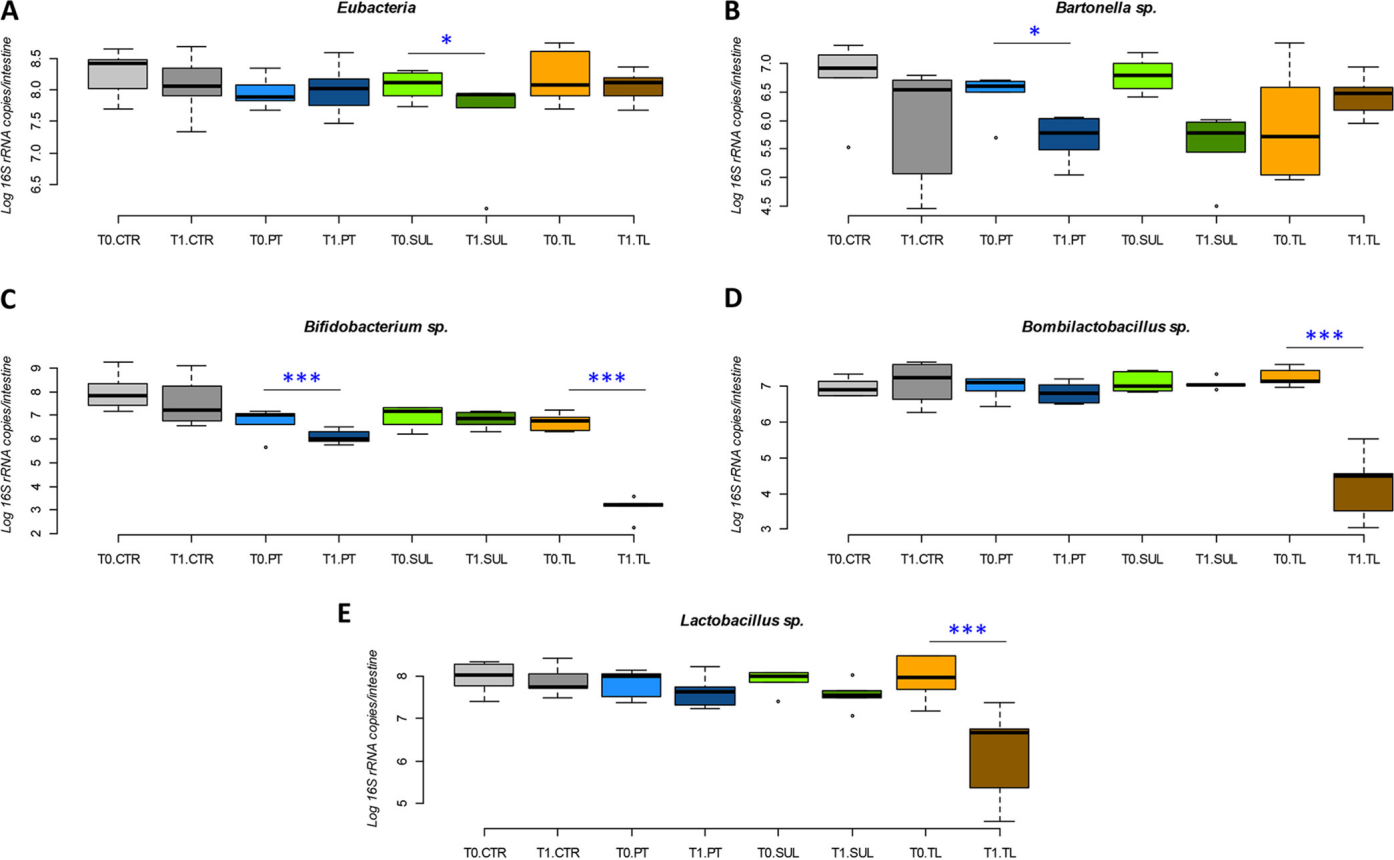
qPCR quantification of total bacteria (Eubacteria) (A), *Bartonella* sp. (B), *Bifidobacterium* spp. (C), *Bombilactobacillus* sp. (D), and *Lactobacillus* sp (E). Data are expressed as the log of 16S rRNA gene copies/intestine for *Bartonella* sp., *Bifidobacterium* spp., *Bombilactobacillus* sp., and *Lactobacillus* sp.; for Eubacteria, data are expressed as the log of 16S rRNA copies/intestine. Boxplots report minimum and maximum values, lower and upper quartile and median. CTR, no antibiotics control; PT, oxytetracycline; SUL, sulfonamides; TL, tylosin.

The counts of Eubacteria (Fig. 1A) at the beginning and at the end of the experiment showed a significant decrease (0.65 log, *P* < 0.05) upon sulfonamide treatment (SUL_T0 versus SUL_T1). Other conditions did not show significant variation. Considering *Bartonella* spp. (Fig. 1B), only PT treatment highlighted a significant decrease between PT_T0 *vs* PT_T1 (0.76 log 16S rRNA copy number decrease, *P* < 0.05). *Bifidobacterium* spp. counts showed a general decrease in all experimental conditions. The reduction was significant in PT_T0 versus PT_T1 (0.58 log 16S rRNA copy number decrease, *P* < 0.01) and in TL_T0 versus TL_T1 (3.61 log 16S rRNA copy number decrease, *P* < 0.01) (Fig. 1C). Also, *Bombilactobacillus* spp. and *Lactobacillus* spp. showed a general decrease in all experimental conditions, which was significant only in the comparison of TL_T0 versus TL_T1 (*P* < 0.01), with a decrease of 2.89 and 1.71 log 16S rRNA copy numbers, respectively (Fig. 1D and E).

Tetracycline resistance genes *tetW* and *tetY* increased significantly by 144% and 180%, respectively, (*P* < 0.01) comparing PT_T0 with PT_T1. Sulphonamides resistance genes *sul1* and *sul2* showed a significant increase (76.84% and 33.95%, respectively, comparing SUL_T1 with SUL_T0, *P* < 0.01), whereas *sul3* could not be amplified at the different annealing temperatures tested (40 to 64°C). Tylosin resistance genes *tlrB* and *tlrD* did not show any significant variation in normalized data. The melting temperature (*T_m_*) of the amplification products immediately after the last reaction cycle and the qPCR efficiency data are reported in Table 1.

**TABLE 1:** Average slope, intercept, *R*^2^, and amplicon *T_m_* of the qPCR performed in this experiment

Taxon or gene	Abbreviation	Avg slope	Avg intercept	*R* ^2^	Amplicon *T_m_* (°C)
*Bartonella* spp.	Bart	3.555	38.806	0.984	74.0
*Bifidobacterium* spp.	Bif	3.509	36.683	0.986	82.0
*Bombilactobacillus* spp.	Firm4	3.599	42.782	0.999	80.0
Eubacteria	Eub	3.549	39.848	0.996	80 ± 1
*Lactobacillus* spp.	Firm5	4.106	47.540	0.998	77.0
Tylosin resistance gene B	*TlrB*	3.914	44.740	0.999	80.1
Tylosin resistance gene D	*TlrD*	3.511	41.345	1.000	81.2
Sulphonamides resistance 1	*Sul1*	3.430	37.551	0.999	87.0
Sulphonamides resistance 2	*Sul2*	3.600	42.685	0.999	87.0
Tetracycline resistance gene W	*TetW*	3.643	36.810	1.000	80.7
Tetracycline resistance gene Y	*TetY*	3.954	35.056	0.999	84.0

### Bee gut microbiota analysis via next-generation sequencing.

**FIG 2:**
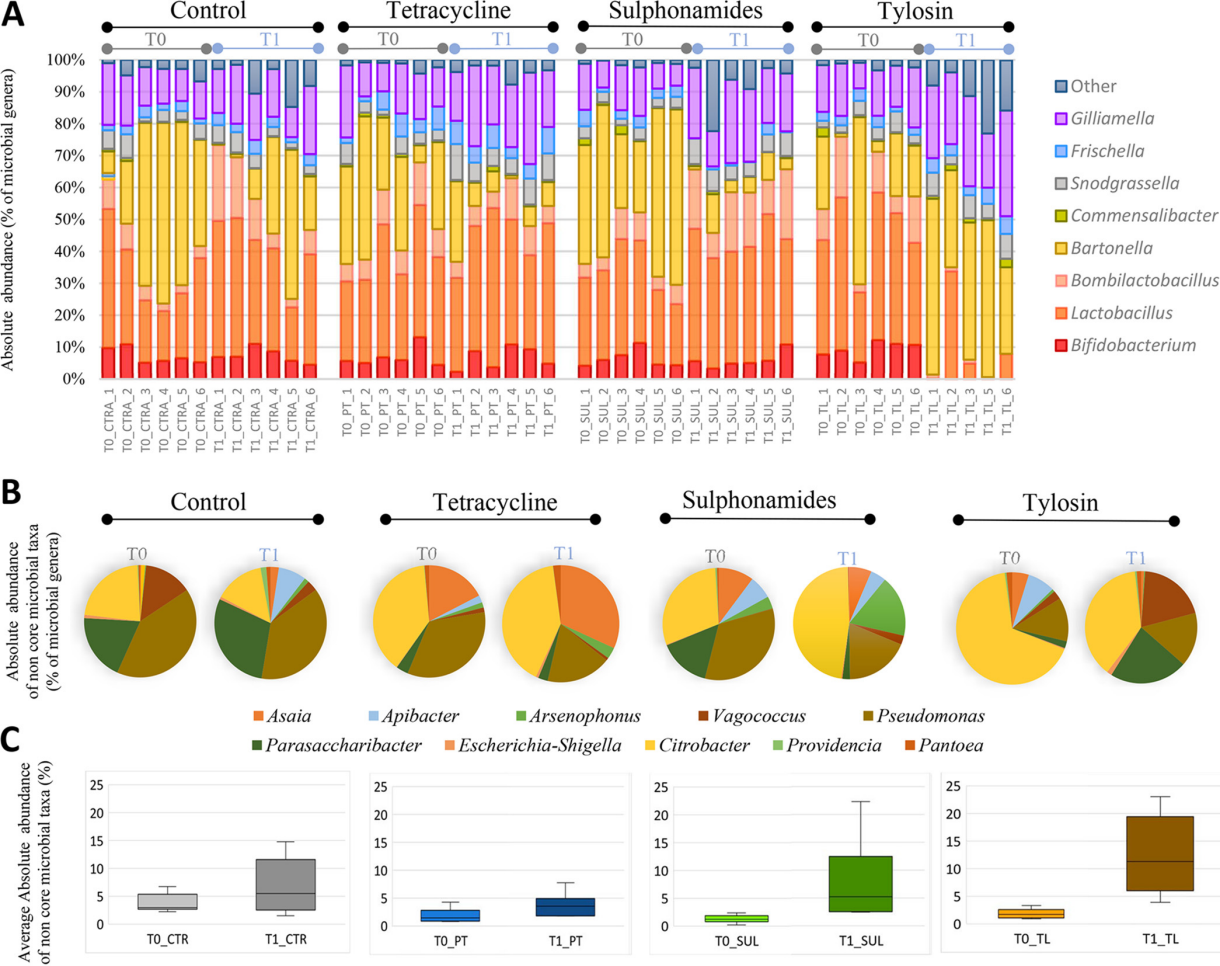
NGS absolute abundance overview. (A) Bar charts reporting the major cumulated microbial genera per samples and their absolute abundance expressed as percentages. (B) Pie charts reporting the minor cumulated microbial genera (Other_taxa) per experimental conditions and sampling time, expressed in percentage as absolute abundance. (C) Average absolute abundance of Other_taxa for each treatment in T0 and T1.

A total of 48 samples (2 sampling times [T0 and T1], 4 experimental conditions [CTR, PT, SUL, and TL], 6 replicates for each condition, each replicate being a pool of 30 honeybee guts) were subjected to next-generation sequencing (NGS) analysis on an Illumina MiSeq platform. About 13.7 million raw reads were obtained from the sequencing. Of these, 9.1 million reads passed the quality control and the chimera check analysis, obtaining an average of 95,986 joint reads per sample. For statistical analysis, samples were rarefied at 48,400 reads, a value obtained with exclusion of one replicate (TL_T1_4) due to a particularly low coverage. The taxonomical assignment of the 47 samples produced 17,194 operational taxonomic units (OTUs) at 97% similarity based on the SILVA 132 database. The obtained NGS data on the whole data set are reported in Table 2, as well as absolute abundance at phyla, families, and genera levels per treatment and time. Figure 2A reports absolute abundance at genus level per replicate.

**TABLE 2:** NGS absolute abundance at phyla, family, and genus level, reported per treatment and sampling time^*a*^

Taxon
	T0_CTR	T1_CTR	T0_PT	T1_PT	T0_SUL	T1_SUL	T0_TL	T1_TL
Phyla								
* Actinobacteria*	7.23	7.27	6.87	6.90	6.32	5.97	9.20	0.02
* Firmicutes*	32.76	45.57	40.94	47.54	34.15	55.21	48.51	12.58
* Proteobacteria*	60.01	47.16	52.18	45.55	59.54	38.82	42.29	87.40
Family								
* Bifidobacteriaceae*	7.23	7.31	6.82	6.65	6.29	5.85	9.12	0.02
* Lactobacillaceae*	32.68	46.10	40.74	45.98	34.02	53.33	48.09	13.16
* Bartonellaceae*	36.12	17.46	26.87	8.86	39.66	5.45	19.18	40.02
* Neisseriaceae*	4.31	3.94	3.94	7.31	3.21	6.07	3.64	5.80
* Acetobacteraceae*	1.58	1.88	1.83	0.90	0.67	5.60	1.89	5.32
* Orbaceae*	15.73	18.68	18.52	26.88	14.46	20.37	16.91	28.53
* *Other_Families	2.36	4.64	1.30	3.44	1.69	3.33	1.18	7.15
Genera								
* Bifidobacterium*	7.23	7.33	6.85	6.64	6.33	5.93	9.32	0.02
* Lactobacillus*	26.94	33.75	32.49	38.57	27.82	37.78	37.52	9.37
* Bombilactobacillus*	5.03	11.72	8.66	7.27	6.13	15.74	10.61	0.81
* Apilactobacillus*	0.00	0.00	0.00	0.00	0.00	0.00	0.00	0.00
* Plantilactobacillus*	0.00	0.00	0.00	0.00	0.00	0.00	0.00	0.03
* Bartonella*	36.44	17.44	26.65	8.91	39.70	5.67	19.18	40.96
* Commensalibacter*	0.53	0.38	0.80	0.68	1.17	0.43	0.86	1.47
* Snodgrassella*	4.32	3.98	4.03	7.36	3.19	6.10	3.65	5.93
* Frischella*	2.46	3.19	4.60	6.01	3.20	0.98	2.84	4.30
* Gilliamella*	13.30	15.43	14.07	20.84	11.18	19.56	14.16	24.72
* *Other_Genera	3.74	6.78	1.84	3.71	1.26	7.81	1.84	12.40

a CTR, control; PT, Pan-Terramicina; SUL, sulphonamides; TL, tylosin; T0, time zero experiment start; T1, first time point.

Detected non-core genera were mainly *Asaia*, *Apibacter*, *Arsenophonus*, *Vagococcus*, Pseudomonas, *Parasaccharibacter*, *Citrobacter*, *Providencia*, and *Pantoea* (Fig. 2B) and their proportions at T0 and T1 are reported in Fig. 2C.

The α-diversity indices (Chao1, observed OTU, and PD whole tree) showed a significant decrease over time only in the tylosin-treated group (*P* < 0.01). The β-diversity indices, considering unweighted UniFrac, underlined statistically significant differences between the CTR and TL treatments. However, considering the abundance of taxa in the weighted UniFrac, not only TL treatment but also SUL treatment resulted in significant differences when compared to CTR.

The intestinal microbial taxa at the different taxonomic levels did not show any significant shift between the two sampling times (T0 and T1) in control bees. A summary of the significant changes, from phyla to species, for each antibiotic treatment over time is reported in Table 3.

**TABLE 3:** Significant variations among microbial groups at phyla, family, genus, and species level according to the experimental conditions^*a*^

Level	SUL	TL	PT	CTR
Phyla	*Firmicutes ↑* *Proteobacteria ↓*	*Actinobacteria ↓ Firmicutes ↓ Proteobacteria ↑*	*Firmicutes ↑*	
Family	*Acetobacteraceae ↑* *Bartonellaceae ↓* *Neisseriaceae ↑* Other_families *↑*	*Bifidobacteraceae ↓* *Lactobacillaceae ↓* *Orbaceae ↑* Other_families *↑*	*Neisseriaceae ↑* *Orbaceae ↑*	
Genus	*Bartonella* ↓ *Bombilactobacillus* ↑ *Frischella* ↓ *Gilliamella* ↑ *Snodgrassella* ↑ Other_genus ↑	*Bartonella* ↑ *Bifidobacterium* ↓ *Bombilactobacillus* ↓ *Gilliamella* ↑ *Lactobacillus* ↓ Other_genus ↑	*Gilliamella* ↑ *Snodgrassella* ↑	
Species	*A. kunkeei* ↑ *Bartonella apis* ↓ *B._mellifer* ↑ *B._mellis* ↑ *Frischella perrara* ↓ *G. apicola* ↑ *S. alvi* ↑	*B. apis* ↑ *B. asteroides* ↓ *B. indicum* ↓ *B. mellis* ↓ *G. apicola* ↑ *L. a*pis ↓ *L. helsinborgensis* ↓ *L. kimbladii* ↓ *L. kullabergensis* ↓ *L. melliventris* ↓	*L. kullabergensis* ↑	

a CTR, control; PT, Pan-Terramicina; SUL, sulphonamides; TL, tylosin.

**FIG 3:**
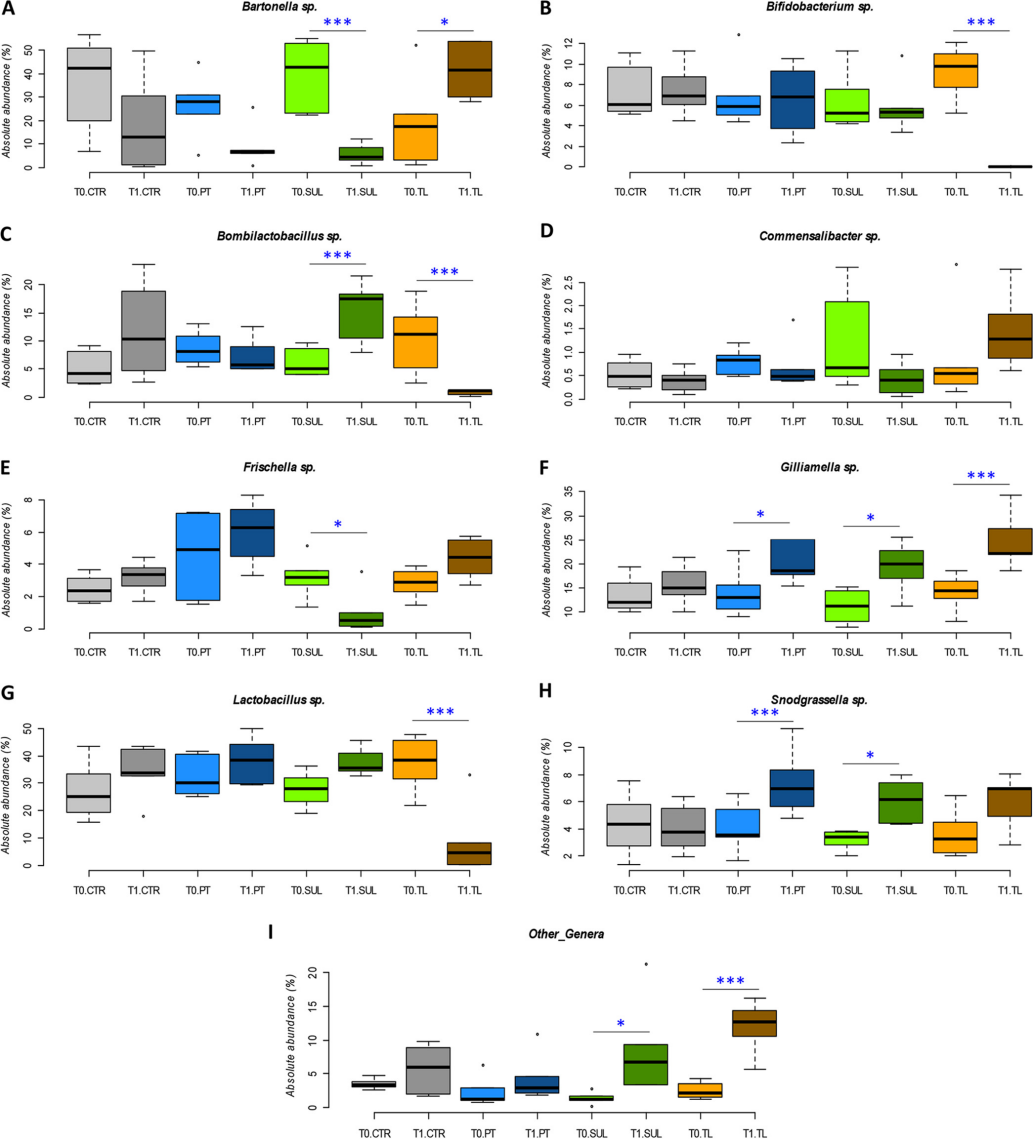
NGS absolute abundance at genus level. (A to F) Box plots reporting the major microbial genera expressed for their absolute abundance (qPCR-normalized NGS relative abundance) in percentage and in relation to experimental conditions (significant pairwise comparisons: *, *P* < 0.05; ***, *P* < 0.01). Boxplots report minimum and maximum values, lower and upper quartile, and median. (A) *Bartonella* spp. (B) *Bifidobacterium* spp. (C) *Bombilactobacillus* spp. (D) *Commensalibacter* spp. (E) *Frischella* spp. (F) *Gilliamella* spp. (G) *Lactobacillus* spp. (H) *Snodgrassella* spp. (I) Other_genera, for the experimental conditions. CTR, no antibiotics control; PT, oxytetracycline; SUL, sulfonamides; TL, tylosin.

The results obtained following PT treatment (comparing PT_T1 versus PT_T0) showed, at phylum level, an increase of *Firmicutes* and a decrease of *Proteobacteria*, although these values were not significant. At family level, both *Neisseriaceae* and *Orbaceae* significantly increased from 3.94% to 7.31% (*P* < 0.01) and from 18.5% to 26.7% (*P* < 0.05), respectively. At genus level, *Gilliamella* spp. almost doubled in absolute abundance (from 14.07% to 20.84%; *P* < 0.05), while *Snodgrassella* spp. significantly increased (from 4.03% to 7.36%; *P* = 0.01) (Fig. 3H). At species level, PT treatment determined a significant increase only for Lactobacillus kullabergensis (*P* < 0.01).

Regarding SUL treatment, at phylum level, *Firmicutes* showed a significant increase comparing SUL_T1 versus SUL_T0 (*P* < 0.05). On the contrary, *Proteobacteria* decreased significantly (*P* < 0.05). At family level, *Bartonellaceae* considerably decreased after treatment (from 39.66% to 5.45%; *P* < 0.01) (Fig. 3C), while *Neisseriaceae* and *Acetobacteraceae* significantly increased (*P* < 0.05). At genus level, SUL treatment resulted in a significant decrease in the absolute abundance of *Bartonella* spp. (*P* < 0.01) (Fig. 3A), and *Frischella* spp. (*P* < 0.05) (Fig. 3E). On the other hand, absolute abundance increased in *Bombilactobacillus* spp. (*P* < 0.01) (Fig. 3C), *Gilliamella* spp. and *Snodgrassella* spp. (*P* < 0.05) (Fig. 3F and H), and Other_genus (*P* < 0.05) (Fig. 3I). At species level, a significant increase was reported for *A. kunkeei* (*P* < 0.05), Bombilactobacillus mellifer (*P* < 0.01), and Bombilactobacillus mellis (*P* < 0.01). Bartonella apis, Frischella perrara, and Gilliamella apicola reflected the genus trend, being the only species within the respective genera.

Regarding tylosin treatment (comparing TL_T1 versus TL_T0), *Proteobacteria* doubled their abundance (*P* < 0.01). On the other hand, both *Firmicutes* and *Actinobacteria* significantly decreased (*P* < 0.01). *Bifidobacteriaceae* and *Lactobacillaceae* significantly decreased comparing TL_T1 and TL_T0 (*P* < 0.01), with percentage values that are consistent with those reported below at the genus level. *Orbaceae* significantly increased at T1 (+68.63%, *P* < 0.01). Finally, the absolute abundance of Other_families significantly increased after TL treatment (+673%, *P* < 0.01). The *Bifidobacterium* spp. absolute abundance reduction after TL treatment was highly significant (*P* < 0.01), decreasing from 9.32% at T0 to 0.02% at T1 (Fig. 3B). In the same way, *Bombilactobacillus* spp. and *Lactobacillus* spp. decreased from 10.61% and 37.52% at T0 to 0.81% and 9.37% at T1 (*P* < 0.01) (Fig. 3C and G), respectively. Moreover, the absolute abundance of *Bartonella* spp. and *Gilliamella* spp. strongly increased (*P* < 0.05) (Fig. 3A and F). Other_genus species significantly increased from 1.84% to 12.40% (*P* < 0.01) (Fig. 3I). At species level, a significant decrease of six *Lactobacillus* species and also of unclassified *Lactobacillus* spp. was observed (*P* < 0.01), together with the decrease of *B. mellis* (*P* < 0.01), Bifidobacterium asteroides (*P* < 0.01), and Bifidobacterium indicum (*P* < 0.05). The Cramer V test showed a strong biological relevance in pairwise comparisons of TL_T1 versus TL _T0 and SUL_T1 versus SUL _T0 (Cramer V = 0.53 and 0.45, respectively) (32). PT_T1 versus PT _T0 and CTR_T1 versus CTR _T0 biological relevance was moderate (Cramer V = 0.25 and 0.23) but not negligible.

**FIG 4:**
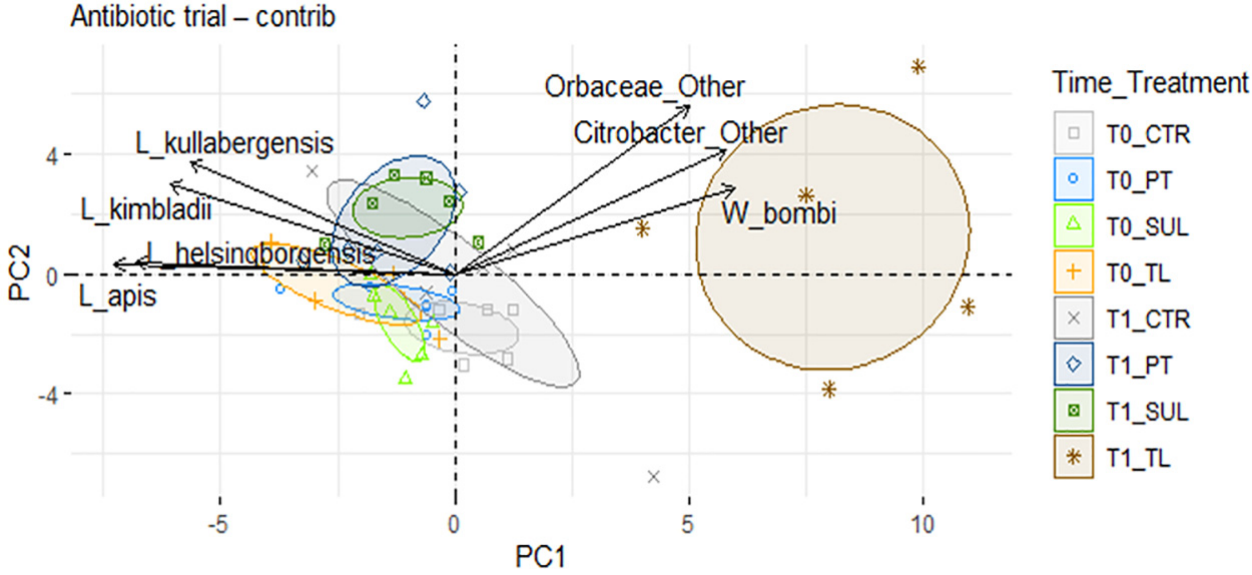
PCA analysis. PCA was performed with 71 taxa at species level; confidence ellipses are shown in the graph. The graph includes the top seven variables with the highest contribution.

Within the principal-component analysis (PCA) of the data set at species level, PC1 and PC2 together explained only 25% of the variability. However, the TL_T1 group is clearly separated from TL_T0 and also from the other treated samples at T1 (Fig. 4A), particularly along the PC1 axis. *Orbaceae* and thus *Gilliamella* spp. are associated with TL_T1, as also confirmed by statistical analysis (Fig. 4B and C). The graph also shows a clear separation of SUL_T0 and T1 along PC2.

## DISCUSSION

In this work, we investigated the gut microbial community of honeybees after the administration of antibiotics (oxytetracycline, sulfonamides, and tylosin) against common bee diseases.

Total bacteria counts were not greatly affected by the antibiotic treatment, whereas the amount of some microbial groups varied significantly upon target antibiotic exposure.

**FIG 5:**
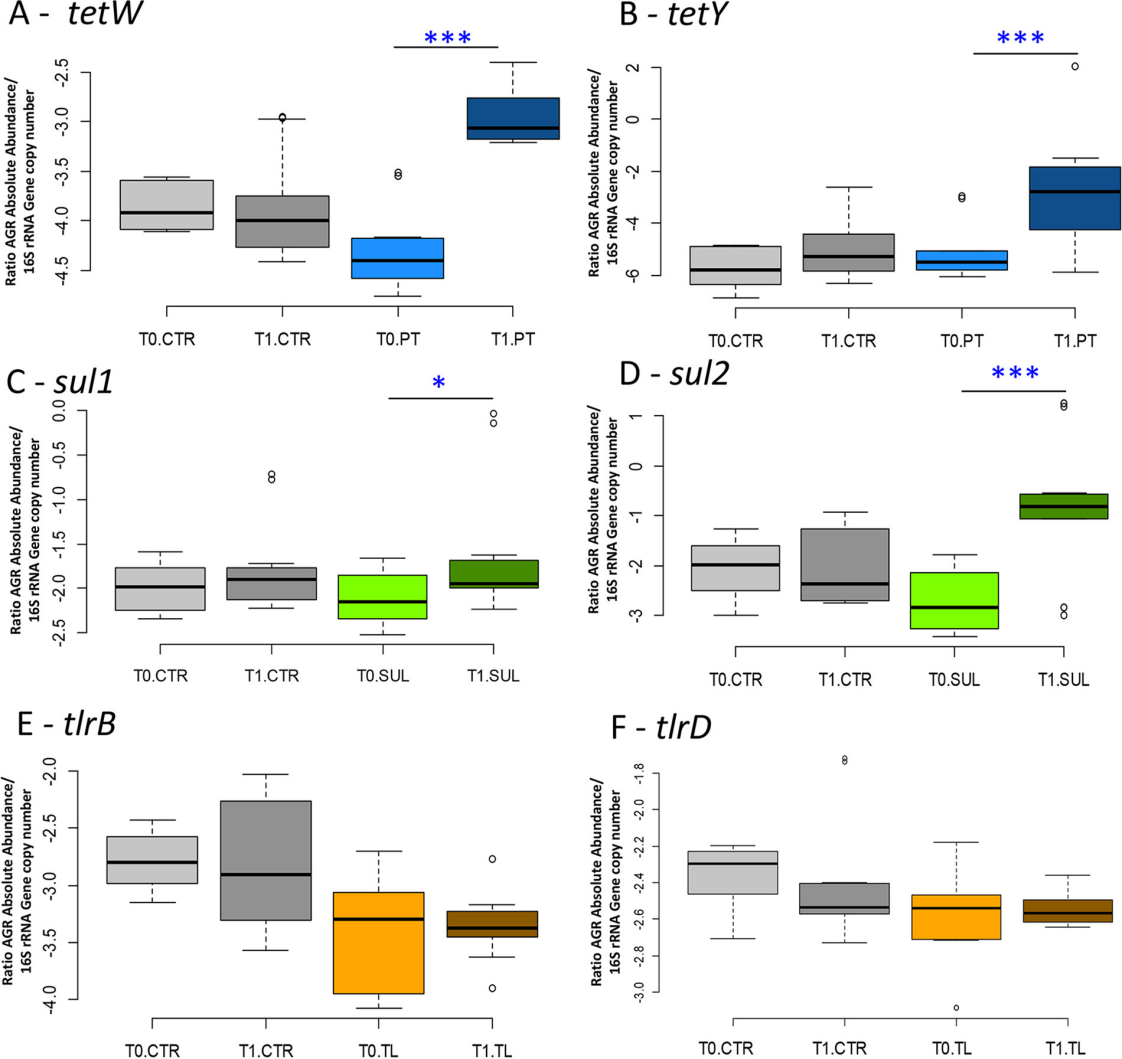
Antibiotic resistance genes. (A and B) Box plots reporting the ARGs for *tetW* (A) and *tetY* (B) for tetracycline resistance genes. (C and D) Box plots reporting the ARGs for *sul1* (C) and *sul2* (D) sulfonamides resistance genes. (E and F) Box plots reporting the ARGs for *tlrB* (E) and *tlrD* (F) tylosin resistance genes. The absolute ARG quantification is normalized with the total 16S rRNA gene copies in relation to experimental conditions (significant pairwise comparisons: *, *P* < 0.05; ***, *P* < 0.01).

Oxytetracycline is a broad-spectrum antibiotic currently used in the beekeeping sector (19, 33). Recently, Raymann et al. (22, 23) showed that the use of tetracycline strongly decreased the absolute abundance of 5 core gut genera in partially caged honeybees, in particular *Bartonella*, *Bifidobacterium*, *Bombilactobacillus* spp. (formerly known as *Lactobacillus* Firm-4), *Lactobacillus*, and *Snodgrassella.* In our study, the large increase of tetracycline-resistance genes in the gut bacteria upon antibiotic treatment is accompanied by the increase of some core members, two of which are significant (*Gilliamella* spp., in agreement with Raymann et al. [22], and *Snodgrassella* spp.). The abundance of other microbial genera, such as *Bartonella* and *Bifidobacterium*, decreased. Our results therefore show an important effect of tetracycline that is focused on specific taxa with minor impact on alfa and beta diversity. It is well known that honeybee gut commensal bacteria provide large reservoirs of tetracycline-resistance determinants (*otr* and *tet* genes) frequently acquired through large and/or long-term antibiotic exposure or from other habitats shared with animals and humans (34, 35). Ludvigsen et al. (35) showed that honeybee gut symbionts, in particular *Snodgrassella* spp. and *Gilliamella* spp., can survive and proliferate thanks to *tet* determinants, and this further supports our results that show a significant increase of these two genera. Recently, Daisley et al. (36) found that the routine administration of oxytetracycline increases *tetW* and *tetY* abundance in the gut microbiota of adult workers and is associated with a depletion of the major symbiont taxa. The present study, therefore, confirms that honeybees may represent a reservoir of tetracycline resistance genes (Fig. 5). In addition, bees, with their daily activities (hive interaction, flying, flower visiting), have a preferred path to integrate their gut microbiota and mitigate the antibiotic damages upon tetracycline administration, as suggested by Daysley et al. (36) for *Lactobacillus* strains. Most of the published studies rely on caged or partially caged honeybees, which limits social behavior as well as interactions with environmental bacteria. In our study, an additional microbial source for the gut microbiota may derive by the reservoir of microbial inoculants within the hive structure (stored pollen, nectar, and wax), which may have contributed to the mitigation of tetracycline impact. Another mechanism that has to be considered in antibiotic resistance genes (ARGs) spreading regards the transmission through horizontal gene transfer (HGT) that has been documented in soil following application of manure containing antibiotic residues and also in human intestine. The HGT of antibiotic resistance genes may allow the increase of ARGs in the studied environment even without major perturbations of the microbiota (37–39).

Sulphonamides (SUL) were widely used in the beekeeping sector from 1960 to 2000, but residues in honey are still found, thus showing they are still used in spite of the banning (40). Among the core genera found in the honeybee gut, *Frischella* and *Bartonella* spp. were significantly affected by SUL treatment, while *Bombilactobacillus* spp. and *Snodgrassella* spp. increased their counts. Frischella perrara has implications in immune priming in honeybees and in the induction of peptides with antimicrobial activity (41). The registered 3% reduction (with a final 1% abundance in T1) may have controversial implications. *F. perrara* reduction could be detrimental for the bee immune stimulation (41), on the other hand, this species has been reported as pathogenic because it causes scab formation in the pylorus (42), therefore *Frischella* reduction might also be positive. *Bartonella* spp. has been related to the recycling of nitrogenous waste products into amino acids and with the degradation of secondary plant metabolites (43). The reduction of more than 80% of this taxon could have implication in digestion functions and in the recovery of amino acids (43). However, it is evident that most of the core members are not affected by SUL treatment. This can again be a consequence of the increase of the sulfonamides-resistant population upon selection after sulfonamides exposure. Accordingly, Cenci-Goga et al. (44) found a high abundance of sulfonamides resistance genes (*sul1* and *sul2*) in honeybees sampled in different Italian locations because of the high SUL spread in the environment.

Tylosin induced a remarkable change in some microbial taxa proportions, almost causing the depletion of the rectum population, in particular of lactobacilli and bifidobacteria, and favoring the hindgut population (mostly *Gilliamella*, but also *Snodgrassella* and *Frischella*). It is known that tylosin targets are mainly Gram-positive bacteria (45, 46). *Bifidobacterium*, *Bombilactobacillus*, and *Lactobacillus* genera represented 99.99% of *Bifidobacteriaceae* and *Lactobacillaceae* family members that, overall, accounted for more than a half of the honeybee gut microbial community. They play an essential role in the transformation of various pollen coat-derived compounds, including flavonoids, phenolamides, and ω-hydroxy acids (47), in addition to the digestion of complex sugars (48, 49). Their rapid decrease may affect honeybee ability to metabolize specific compounds and consequently reduce nutrient availability. It is interesting to report that the macrolide antibiotic resistance genes *tlrB* and *tlrD* did not increase significantly in treated honeybees at T1, even if detected. This is probably due to the low occurrence of these ARGs in *Bombilactobacillus*, *Lactobacillus*, and *Bifidobacterium* honeybee strains, even if TL-resistant strains have been described in humans and swine (50, 51). *Tlr* genes belong to the same resistance group as *erm* genes (erythromycin ribosome methylation), so that *tlrB* is also classified as *erm32* and *tlrD* as *ermN* (52, 53). The maintenance of *tlr* gene abundance may also be explained by their activity against other macrolide antibiotics with a broader spectrum of activity, including Gram-negative bacteria that survived the TL treatment. Indeed, Jackson et al. (54) found that *erm* genes can be activated after tylosin use.

Several studies showed that environmental species, such as members of the *Asaia*, *Apibacter*, *Apilactobacillus*, *Vagococcus*, Pseudomonas, *Parasaccharibacter*, *Citrobacter*, *Providencia*, and *Pantoea* genera, often related with soil, pollen, and nectar (55, 56), are present in the honeybee gut as minor groups (57–59). These non-core genera were found to increase at T1 upon treatments with SUL and TL. These microorganisms may promote the increase of the pool of ARGs due to their continuous exposure to antibiotics used in agriculture, such as the use of sewage from livestock as a soil amendment. Among these strains, Parasaccharibacter apium, recently reclassified as *Bombella* sp. by Smith et al. (60), is reported as a strong immune-stimulating strain in honeybees, also capable of counteracting *Nosema* sp. (61). Therefore, the non-core genera that are sporadically associated with honeybees might play a role in the immune stimulation or metabolic regulation of honeybees, despite their low abundance, and may increase upon antibiotic treatment.

Overall, the three assayed veterinary drugs seem not to influence the total amount of bacteria but rather the absolute abundance of several core and non-core taxa, causing a possible lack of metabolic functions related to the most susceptible bacterial species and strains. A long-term observation of the colony health status, also including the hive development and hive products (e.g., honey), will allow the understanding of the relationship between the altered microbial structure and the behavior and performance of honeybees.

## MATERIALS AND METHODS

### Experimental design.

Due to the European and national laws restricting the use of antibiotics or other veterinary drugs in the open field, tests were conducted in semi-field conditions, i.e., in micro-hives incubated in a thermostatic chamber with a short flying time for honeybees. Honeybees employed in this study had not been treated with antibiotics for several generations (over 2 decades).

**FIG 6:**
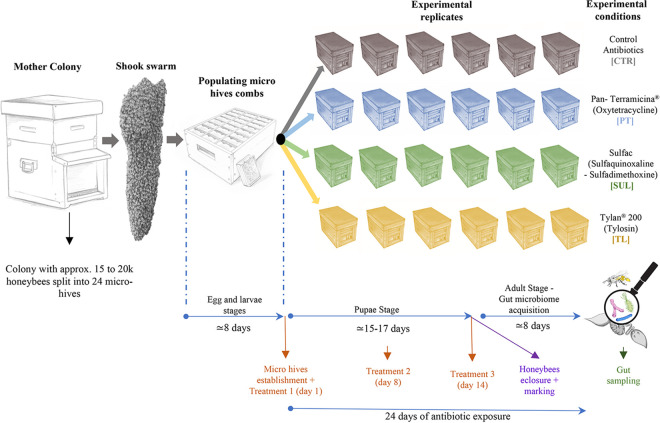
Experimental design. The figure reports the scheme of the tests and the number of bees and beehives used in the trials.

The micro-hives employed in the study were obtained as depicted in Fig. 6. A number of wax combs obtained from a fully populated and healthy bee colony were shaken on a box containing 72 new micro-combs (L 9.5 × H 10.5 cm), causing the fall of thousands of honeybees on the provided micro-frames and populating them (this procedure is referred to as the “shook swarm” method). The queen was allowed to lay eggs for 3 days on approximately 1/3 of the total available micro-combs. Five days later, 24 experimental wooden micro hives (L 20 × H 15 × W 16 cm) were set up, each containing 3 micro-combs (a brood frame, a honey frame, and an empty comb). Each micro-hive contained approximately 500 honeybees with a mated queen. The obtained micro-hives constituted the experimental replicates (6 for each experimental condition). Moreover, every micro-hive was equipped with an anti-robbing entrance modification, forcing honeybees to cover an “S” path that discouraged the entrance of robber bees when the micro hives were placed outside.

Micro-hives were placed into an incubator with controlled temperature and humidity (29°C and relative humidity [RH] of 60), and equipped with a net allowing ventilation on the mini-hive bottom. The micro-hives were moved outside in the late afternoon (approximately from 5:30 p.m. to 8:30 p.m.) every second day in order to allow the bees to fly freely and defecate. The arrangement of the micro-hives outdoors in the experimental field always followed the same pattern to avoid disorientation and drift. Micro-hives were placed at minimum 2 m distance from each other, and in clusters of 3 units of the same experimental thesis, oriented in different directions, in an experimental forest well populated by trees.

At evening, micro-hives were closed and relocated to the lab incubator. Micro-hives were fed every 2 days with 30 ml 1:1 (wt/wt) sucrose solution, plus a dispenser containing 5 ml sterile water. The day of the antimicrobial treatment, honeybees were treated as described below. The developed experimental conditions were: TL, tylosin; PT, oxytetracycline; SUL, a mixture of sulfaquinoxaline and sulfadimethoxine; and CTR, the control with no antibiotic administration. Details on antibiotic use and concentrations are reported below.

The trial was carried out between July and August 2016, where two foraging options were available: (i) honeydew made by the planthopper Metcalfa pruinosa in early august and (ii) Medicago sativa (alfalfa) blooming all through the trial, even if strongly limited by summer drought. The health status (adult honeybee population and brood size, honey reserves, core colony cohesion, symptoms of viral diseases, and varroa infestation) of honeybee micro-hives was periodically assessed, and variations annotated when relevant.

### Treatment preparation, administration, and sampling.

Antibiotics were administered according to available guidelines for each antibiotic (62–64). Details and concentrations of antibiotics are reported in Table 4. Bees were treated once a week for a total of three treatments with micro-hive feeders containing 30 ml of sugar syrup (1:1 wt/wt) mixed with the respective treatment. Finally, after the 3rd treatment (days 15 to 17), at least 50 emerging honeybees per replicate were marked on the thorax (65) with colored nail polish nontoxic to bees. Marked honeybees were sacrificed at day 24, at nurse stage (7 to 9 days post eclosure), and with a completely established gut microbiota (66). A pool of 30 bees per replicate (a total of 180 samples/experimental condition) was picked at the beginning of the experiment (T0) and after 24 days (T1).

**TABLE 4:** Antibiotics used in this work, their dosages applied in each treatment per hive in the presented trials, and recommended doses for full-size colonies^*a*^

Experimental theses			Dose per treatment (mg)^*b*^	Recommended dose for full-size colonies^*c*^	Reference
Treatment	Active ingredient	Commercial brand			
CTR	NA	NA	NA	NA	NA
PT	Oxytetracycline HCl	Pan-Terramicina Zoetis	13.5	800–1200 mg	(62, 63)
SUL	Sulfaquinoxaline 2% + Sulfadimethoxine 1%	Sulfac Formevet	4.5	1 g/3.7 liter	(63)
TL	Tylosin Tartrate	Tylan Soluble Elanco	10.0	200 mg/7 g powdered sugar	(64)

a CTR, control; PT, Pan-Terramicina; SUL, sulphonamides; TL, tylosin; NA, not applicable. All antibiotics or antimicrobial agents were prepared in 30 ml of sugar syrup and sprayed on. or fed to bees.

b Dose recalculated according to the colony size of micro-hives, expressed as mg or μl of active ingredient dissolved in 30 ml of sugar syrup.

c Total recommended dose for 3 administrations with weekly cadence.

### DNA extraction and NGS sequencing.

Obtained honeybee gut pools were well homogenized with a pestle, after which was added 1,400 μl of lysis solution containing 60 μl proteinase K per pool (20 mg/ml concentration) and glass beads. Total destruction of gut epithelial tissues was obtained after 1 h incubation at 55°C. Only 1/4 of the resulting sludge (450 μl) was used for gut genomic DNA extraction with Quick-DNA Fecal and Soil Microbe kit (Zymo Research, California, USA). The 16S rRNA gene amplification and libraries prepared for Illumina MiSeq platform sequencing were carried out according to Alberoni et al. (67). Briefly, the V3-V4 was amplified with KAPA Hi-Fi PCR Master Mix (Roche, Monza, Italy) with a maximum of 25 cycles. PCR products were purified with AMPure magnetic beads (Beckman Coulter, Milan, Italy) and indexed with i7 and i5 Illumina adapters (Illumina, Milan, Italy). NGS sequencing was performed with the addition of 22% PhiX to the sample pool (Illumina, Milan, Italy). Bioinformatic analyses were performed with Qiime1, and representative operational taxonomic units (OTUs) were subjected to BLAST search against the most updated SILVA database release 132. OTUs with less than 0.1% abundance were discarded. The α–diversity was evaluated using Chao1, observed OTU, and PD whole tree metrics, whereas β–diversity was evaluated using both weighted and unweighted UniFrac.

### Quantification of target microbial groups and resistance genes.

The main microbial groups found in the honeybee rectum (*Bartonella* spp., *Bifidobacterium* spp., *Bombilactobacillus* spp., and *Lactobacillus* spp.), as well as total bacteria (Eubacteria) were quantified with qPCR (StepOne real-time PCR system, Applied Biosystems) according to Baffoni et al. (68, 69). Briefly, standard curves were constructed using PCR products of the 16S rRNA gene for the target microbial genera. The PCR products were purified and serially diluted to obtain standards ranging from 10^4^ to 10^8^ gene copies. Amplification reactions were performed on a total volume of 20 μl using the Fast SYBR Green Master Mix (Applied Biosystems). Moreover, DNA quantity was standardized at 5 ng/μl of DNA. The specificity of the reaction given by the *T_m_* of the amplification products is reported in Table 1.

Absolute quantification of the target microbial groups was obtained by multiplying the absolute quantification data with the total extracted DNA and then divided by the gut number and the gene copy number (except for Eubacteria) (70, 71). The data was expressed in log 16S rRNA copies/intestine (72). ARGs *TetW*, *TetY*, *Sul1*, *Sul2*, *Sul3*, *TlrB*, and *TlrD* (Fig. 5) were quantified according to Zhang et al. (73). The primers used are reported in Table 5. Raw data were corrected according to the total DNA quantification. The final absolute abundance of ARGs was normalized according to references 74 and 75 by dividing the total ARGs with the absolute abundance of total bacteria previously obtained.

**TABLE 5:** List of primers used in this experiment to carry out quantification of specific microbial targets and detection of ARGs

Taxon or gene	Primer name	Sequence (5′–3′)	Amplicon size	Reference
*Bartonella* spp.	Bart-F	GTGGGAATCTACCTATTTCTACG	103	(30)
	Bart-R	AACGCGGGCTCATCTATCTC		
*Bifidobacterium* spp.	Bif TOT-F	TCGCGTCYGGTGTGAAAG	243	(82)
	Bif TOT-R	CCACATCCAGCRTCCAC		
*Bombilactobacillus* spp.	Firm4-F	AGTCGAGCGCGGGAAGTCA	169	(30)
	Firm4-R	AGCCGTCTTTCAACCAGCACT		
Eubacteria	Eub338-F	ACTCCTACGGGAGGCAGCAG	200	(83)
	Eub518-R	ATTACCGCGGCTGCTGG		
*Lactobacillus* spp.	Firm5-F	GCAACCTGCCCTWTAGCTTG	118	(33)
	Firm5-R	GCCCATCCTKTAGTGACAGC		
Tylosin resistance gene B	Tlr B-F	GTGTCCTGGAGGAGTTCGAG	111	(83)
	Tlr B-R	AGCGGAAGTGTGTCCCATAC		
Tylosin resistance gene D	Tlr D-F	GTCAACGACGACTTCACGAC	186	(83)
	Tlr D-R	ACTGGGCGTTGAAGAGATTG		
Sulphonamides resistance 1	Sul1-F	CGGCGTGGGCTACCTGAACG	433	(84)
	Sul1-R	GCCGATCGCGTGAAGTTCCG		
Sulphonamides resistance 2	Sul2-F	GCGCTCAAGGCAGATGGCATT	293	(84)
	Sul2-R	GCCTTTGATACCGGCACCCGT		
Sulphonamides resistance 3	Sul3-F	TCCGTTCAGCGAATTGGTGCAG	/	(85)
	Sul3-R	TTCGTTCACGCCTTACACCAGC		
Tetracycline resistance gene W	TetW-F	GAGAGCCTGCTATATGCCAGC	168	(86)
	TetW-R	GGGCGTATCCAGAATGTTAAC		
Tetracycline resistance gene Y	TetY-F	GCTGATATTTGCGGGTTTCTA	177	(87)
	TetY-R	CGTCAAGCCTGTTAAAGTTCC		
Illumina adapter - V3-V4 Region of 16S rRNA gene	Pro341-F	*AATGATACGGCGACCACCGAGATCTACACTCTTTCCCTACACGACGCTCTTCCGATCTCCTACGGGAGGCAGCAG*-CCTACGGGNGCASCAG	560	(88)
	Pro805-R	*CAAGCAGAAGACGGCATACGAGATNNNNNNGTGACTGGAGTTCAGACGTGTGCTCTTCCGATCT*-GACTACNVGGGTATCTAATCC		

### Data adjustments and classification of microbial genera.

Rarefied biom tables obtained from NGS bioinformatic analysis were used for further data adjustments, where the absolute abundance of each bacterial species was calculated according to Raymann et al. (22) by multiplying absolute abundance data to the corresponding qPCR total amount results, and then normalizing by the copy number of the 16S rRNA gene typical of each microbial genus. Moreover, species belonging to the *Lactobacillus* genus have been recently reclassified (76) but databases for NGS OTUs assignment were not yet updated with the new classification at the time of the bioinformatic analysis of the presented data. Therefore, the data set was manually adjusted according to Alberoni et al. (77) in order to reassign former *Lactobacillus* sp. Firm-4 to the *Bombilactobacillus* spp. genus and the former Lactobacillus kunkeei and Lactobacillus plantarum to the new respective taxonomical classifications Apilactobacillus kunkeei and Lactoplantibacillus plantarum. Due to the concern that sequencing amplicon length (≈470 bp) might not be enough to efficiently discriminate among species, manual curation was then used to validate by qPCR with Firm-4- and Firm-5-specific primers (30). The obtained data set was used for further graphical and statistical analyses on target genera and species.

### Compliance with ethical standards.

This article does not contain any studies with human participants by any of the authors and experiments on animals were performed according to the Italian laws allowing experiments on arthropods without the need of an official ethical commission approval, unless cephalopods are used.

### Statistical analysis.

Statistical analysis for qPCR and NGS data (α-diversity and taxon analysis) was performed with the R software (78) according to Baffoni et al. (68). Analysis on data normality and homoscedasticity was performed and normal and homoscedastic data were analyzed with ANOVA; nonnormal homoscedastic data (with normal distribution of residuals) were analyzed with glm function, while data with high deviation from normality were analyzed with the nonparametric Kruskal-Wallis test coupled with the Dunn test. For β–diversity index, data resulting from QIIME statistical elaboration were reported. The software calculates the UniFrac distance (weighted and unweighted UniFrac) between all the pairs of samples in the data set to create a distance matrix. The statistical significance between groups was subsequently estimated using the Monte Carlo method with the Bonferroni correction.

*Post hoc* tests among different groups were carried out and Bonferroni’s correction was applied. The *post hoc* test considered pairwise comparisons within each experimental condition, taking into consideration the impact of each treatment over time. Therefore, four comparisons for the semi-field trial and three comparisons for the in-field trial were considered. The control was considered as a further treatment to monitor and evaluate the normal gut microbial community evolution resulting from the interaction of honeybees with the environment. Graphs were generated with ggplot2, ggpubr, and Microsoft Excel. The biological relevance of experimental conditions, pairwise compared at their respective sampling time (T1 versus T0), was computed with Cramér’s V (79) relying on packages rcompanion, vcd, psych, desctools, and epitools. Finally, PCA analysis was performed using packages FactoMineR (80) and factoextra (81), taking into consideration 71 taxa at species level.

### Data availability.

These sequence data have been submitted to the NCBI repository Sequence Read Archive (SRA) databases under accession numbers SAMN16442373 to SAMN16442378; SAMN16442391 to SAMN16442396; SAMN16442397 to SAMN16442402; SAMN16442409 to SAMN16442414; SAMN16442427 to SAMN16442432; SAMN16442444 to SAMN16442449; SAMN16442450 to SAMN16442455, and SAMN16442462 to SAMN16442467, with BioProject number PRJNA669646. Supplemental data, including Exel files of elaborated data obtained from qPCR for target microbial groups and ARGs and NGS data categorized at phyla, family, and genera levels, are available on reasonable request from the corresponding author.
